# The frequency and clinical presentation of Zika virus coinfections: a systematic review

**DOI:** 10.1136/bmjgh-2020-002350

**Published:** 2020-05-07

**Authors:** Ludmila Lobkowicz, Anna Ramond, Nuria Sanchez Clemente, Ricardo Arraes de Alencar Ximenes, Demócrito de Barros Miranda-Filho, Ulisses Ramos Montarroyos, Celina Maria Turchi Martelli, Thalia Velho Barreto de Araújo, Elizabeth B Brickley

**Affiliations:** 1 Department of Infectious Disease Epidemiology, London School of Hygiene and Tropical Medicine, London, UK; 2 Departamento de Medicina Interna, Universidade de Pernambuco, Recife, Pernambuco, Brazil; 3 Instituto de Ciências Biológicas, Universidade de Pernambuco, Recife, Pernambuco, Brazil; 4 Instituto Aggeu Magalhães, Fundação Oswaldo Cruz, Recife, Pernambuco, Brazil; 5 Departamento de Medicina Social, Universidade Federal de Pernambuco, Recife, Pernambuco, Brazil

**Keywords:** systematic review, arboviruses, epidemiology

## Abstract

**Background:**

There is limited knowledge on the influence of concurrent coinfections on the clinical presentation of Zika virus (ZIKV) disease.

**Methods:**

To better understand the types, frequencies and clinical manifestations of ZIKV coinfections, we did a systematic review of four databases (PubMed, Embase, Web of Science, LILACS) without restrictions for studies on ZIKV coinfections confirmed by nucleic acid (quantitative real-time-PCR) testing of ZIKV and coinfecting pathogens. The review aimed to identify cohort, cross-sectional, case series and case report studies that described frequencies and/or clinical signs and symptoms of ZIKV coinfections. Conference abstracts, reviews, commentaries and studies with imprecise pathogen diagnoses and/or no clinical evaluations were excluded.

**Results:**

The search identified 34 articles from 10 countries, comprising 2 cohort, 10 cross-sectional, 8 case series and 14 case report studies. Coinfections were most frequently reported to have occurred with other arthropod-borne viruses (arboviruses); out of the 213 coinfections described, ZIKV infections co-occurred with chikungunya in 115 cases, with dengue in 68 cases and with both viruses in 19 cases. Other coinfecting agents included human immunodeficiency, Epstein-Barr, human herpes and Mayaro viruses, *Leptospira* spp, *Toxoplasma gondii* and *Schistosoma mansoni*. ZIKV-coinfected cases primarily presented with mild clinical features, typical of ZIKV monoinfection; however, 9% of cases in cohort and cross-sectional studies were reported to experience complications.

**Conclusion:**

Based on the evidence collated in this review, coinfections do not appear to strongly influence the clinical manifestations of uncomplicated ZIKV infections. Further research is needed to confirm whether risk of severe complications is altered when ZIKV infection co-occurs with other infections.

**PROSPERO registration number:**

CRD42018111023.

**Key questions**

**What is already known?**

- As Zika virus (ZIKV) has been most prevalent in subtropical and tropical regions with high burdens of cocirculating infectious agents, a proportion of ZIKV infections occur simultaneously with infections by one or multiple other pathogens; however, it is uncertain whether coinfections may influence ZIKV-related pathology.

**What are the new findings?**

- This systematic review collated the evidence on ZIKV coinfections as published in 34 studies in 10 countries. ZIKV coinfections were most frequently reported in the context of the arthropod-borne viruses, dengue and chikungunya, but were also described in relation to eight other pathogens.
- While the findings of this review suggest that coinfections do not appear to strongly influence the clinical manifestations of uncomplicated ZIKV infections, this review did identify reports of neurological complications in the context of coinfection.

**What do the new findings imply?**

- The findings of this review highlight a need for coordinated and rapid research efforts during future outbreaks to optimise diagnostic testing strategies for detecting coinfections and determining whether they may exacerbate the risk of severe ZIKV complications, such as Guillain-Barré syndrome and congenital Zika syndrome.

## Introduction

Zika virus (ZIKV) is an *Aedes* mosquito-borne flavivirus that recently emerged in the Americas.^1^ First recognised in Brazil in early 2015, the ZIKV epidemic spread explosively, with autochthonous transmission reported in more than 86 countries and territories by 2018.^1^ Given the widespread circulation of this emerging infection of public health concern, it is critical that healthcare practitioners can readily recognise ZIKV disease across the full range of its clinical presentations.

Current evidence indicates that ZIKV infections typically present with no or mild clinical features.^1^ A 2018 meta-analysis of 23 studies by Haby and colleagues estimated a prevalence of asymptomatic ZIKV infections of 62% (95% CI 33% to 87%).^2^ For symptomatic ZIKV disease, the WHO describes a mild clinical presentation marked by fever, rash, conjunctivitis, myalgia, arthralgia, malaise and headache.^1^ Nevertheless, ZIKV is neurotropic and, in a subset of cases, infections have been associated with severe neurological complications, including the polyneuropathy Guillain-Barré syndrome (GBS) and congenital Zika syndrome (CZS), a constellation of congenital central nervous system malformations resulting from the vertical transmission of ZIKV during pregnancy.^3^ It has been estimated that GBS arises in approximately 2 per 10 000 ZIKV infections,^1 4^ and the absolute risk of adverse birth outcomes (ie, miscarriage, stillbirth, premature birth and CZS) has been reported to range between 7% and 46% in pregnancies with quantitative real-time PCR (qRT-PCR)-confirmed ZIKV infection.^5–8^

Although the clinical presentation of ZIKV monoinfections has been well characterised, one factor that may influence the clinical spectrum of ZIKV disease is coinfection. Given the high incidence of infectious diseases in the subtropical and tropical areas where ZIKV is prevalent, a proportion of all ZIKV infections occur concurrently with infections by one or multiple pathogens.^9^ ZIKV disease in the context of coinfection remains inadequately investigated, and it is uncertain whether specific coinfections may influence the presentation and severity of ZIKV-related signs and symptoms. A 2019 literature review by Vogels and colleagues hypothesised that coinfecting agents have the potential to enhance, inhibit, compete with or have no effect on ZIKV replication and the resulting clinical disease.^10^ To advance understanding on this topic, this systematic review aims to quantify how frequently ZIKV coinfections occur among ZIKV-infected populations and to investigate whether the clinical course of ZIKV disease in humans is altered in the context of coinfection.

## Methods

### Search

Four databases (PubMed, Web of Science, LILACs and EMBASE) were searched for publications up to 19 October 2019 using a comprehensive search strategy (online supplementary appendix 1). Keywords and Medical Subject Headings linked to ZIKV, bacterial, parasitic and other viral infectious diseases were used. The search included English, French, Spanish and Portuguese terms. No date or language restrictions were applied. The systematic review was registered in PROSPERO. All study titles and abstracts were screened based on eligibility criteria, and references of included studies were also screened to identify additional eligible articles.

10.1136/bmjgh-2020-002350.supp1 Supplementary data

### Study selection and data extraction

Cohort studies, cross-sectional studies, case series and case reports describing coinfections of ZIKV with one or multiple other pathogens, confirmed by nucleic acid testing (eg, qRT-PCR) for ZIKV, and all coinfecting pathogens were eligible for inclusion in the review. Recovery of live pathogens was also considered to be indicative of acute coinfection. Of note, HIV-positive ZIKV cases with HIV suppression were not included in this review. Two reviewers (AR and LL) simultaneously screened studies for eligibility, and any discrepancies were resolved by a third reviewer (EBB). Conference abstracts, reviews, commentaries and studies without nucleic acid confirmation were excluded. Whereas cohort, cross-sectional and case series studies reporting on numbers of ZIKV coinfections without description of signs and symptoms were included to describe the frequency of ZIKV coinfections, studies with no reporting of signs and symptoms of ZIKV coinfections were otherwise excluded from the review. Data extraction was independently performed by two reviewers (AR, LL). From the full-text articles, information on study author, location, year, data source, age and sex of identified cases was extracted. Additional extracted information included frequencies of ZIKV cases with coinfection, types of coinfection, types of diagnostic testing, reported signs and symptoms, non-infectious comorbidities, and types and frequencies of complications. To investigate the frequency of ZIKV coinfections in cohort, cross-sectional and case series studies, the numbers of coinfections out of the total number of qRT-PCR-confirmed ZIKV cases were calculated for the eligible studies. The study quality assessment was conducted using the Oxford Centre for Evidence-based Medicine (OCEBM) Levels of Evidence, March 2009^11^; see online supplementary appendix 2 for details.

### Patient and public involvement

This research was done without patient or public involvement.

## Results

### Study selection

**Figure 1:**
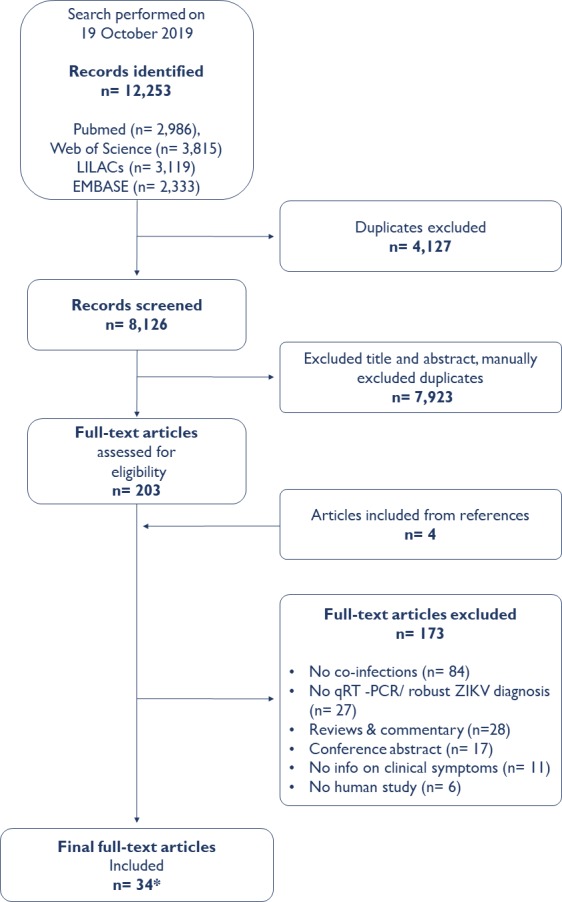
Study selection.

**Figure 2:**
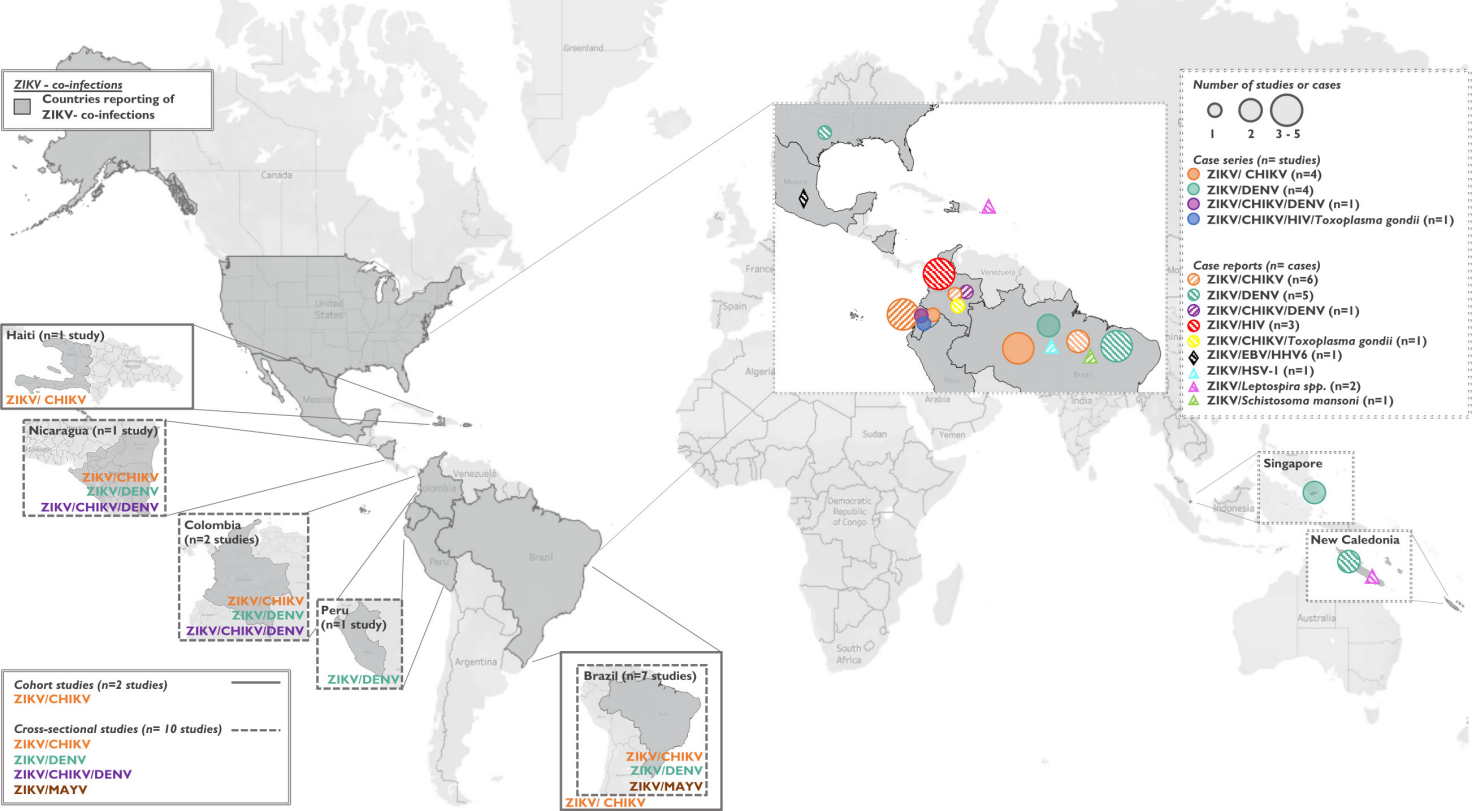
Studies included in the systematic review: cohort studies (n=2), cross-sectional studies (n=10), case series studies (n=8) and case reports (n=21 reported in 14 case report studies). Two cohort studies on ZIKV/CHIKV coinfections were conducted in Haiti (n=1) and Brazil (n=1). Ten cross-sectional studies were conducted in Brazil (n=6), Colombia (n=2), Nicaragua (n=1) and Peru (n=1). Eight case series were reported from Brazil (n=5), Ecuador (n=1) and Singapore (n=2). Twenty-one case reports were reported from Brazil (n=6), Colombia (n=6), Ecuador (n=3), Mexico (n=1), New Caledonia (n=3), Puerto Rico (n=1) and the USA (n=1). CHIKV, chikungunya virus; DENV, dengue virus; EBV, Epstein-Barr virus; HSV, herpes simplex virus; MAYV, Mayaro virus; ZIKV, Zika virus.

The search initially identified 12 253 titles, of which 12 050 titles were excluded after screening titles and abstracts and removing duplicates (figure 1). Full-text screening was completed for 203 publications, and, ultimately, 34 articles representing coinfections in 10 countries were included (tables 1–4 and figure 2).

*Studies reporting on both clinical signs and symptoms and frequency of Zika virus coinfections (n=27); studies reporting only on Zika virus coinfection frequencies (n=7).qRT-PCR, quantitative real-time PCR; ZIKV, Zika virus.

**Table 1:** ZIKV/CHIKV, ZIKV/DENV and ZIKV/CHIKV/DENV coinfection frequencies among qRT-PCR-confirmed ZIKV infected study population (n=11 studies)

Author (year)	Country/ Territory	Region	Study year	Study design	Study population*	Coinfecting agent(s)	Coinfection cases (n)	qRT-PCR-confirmed ZIKV infected study population (n)	Frequency (%)	Level of evidence†
Mercado-Reyes *et al*^28^ (2018)	Colombia	N/A‡	Oct 2015– Dec 2016	Cross sectional	Suspected arbovirus infections	CHIKV	28	10 118	0.3%	2c
Brasil *et al*^5^ (2016)	Brazil	South-East	Sept 2015– May 2016	Cohort	Pregnant women with rash	CHIKV	3	182	1.7%	2b
Magalhaes *et al*^12^ (2017)	Brazil	North-East	May 2015– May 2016	Cross sectional	Suspected arbovirus infections	CHIKV	2	26	7.7%	2c
Waggoner *et al*^13^ (2016)	Nicaragua	N/A	Sept 2015– Apr 2016	Cross sectional	Suspected arbovirus infections	CHIKV	16	75	21.3%	2c
Carrillo- Hernández *et al*^9^ (2018)	Colombia	East	Aug 2015– Apr 2016	Cross sectional	Suspected arbovirus infections	CHIKV	10	29	27.6%	2c
Charlys da Costa *et al*^14^ (2017)	Brazil	North-East	Mar 2016– May 2016	Cross sectional	Suspected arbovirus infections with rash	CHIKV	36	66	54.0%	2c
Mercado-Reyes *et al*^28^ (2018)	Colombia	N/A‡	Oct 2015– Dec 2016	Cross sectional	Suspected arbovirus infections	DENV	3	10 118	0.03%	2c
Chia *et al*^15^ (2017)	Singapore	Singapore	Aug 2016– Sept 2016	Case series	Suspected ZIKV infections	DENV	4	163	2.4%	4
Pessôa *et al*^16^ (2016)	Brazil	North-East	May 2015	Case series	Suspected arbovirus infections	DENV	1	31	3.2%	4
Colombo *et al*^17^ (2017)	Brazil	South-East	Jan 2016–Nov 2016	Cross sectional	Suspected ZIKV infections	DENV	4	100	4.0%	2c
Estofolete *et al*^18^ (2018)	Brazil	South-East	Jan 2016–Nov 2016	Case series	Suspected arbovirus infections	DENV	12	151	7.9%	4
Waggoner *et al* (2016)^13^	Nicaragua	N/A	Sept 2015 –Apr 2016	Cross sectional	Suspected arbovirus infections	DENV	6	75	8.0%	2c
Carrillo- Hernández *et al*^9^ (2018)	Colombia	East	Aug 2015– Apr 2016	Cross sectional	Suspected arbovirus infections	DENV	12	29	34.4%	2c
Azeredo *et al*^19^ (2018)	Brazil	Central-West	Feb 2016– Mar 2016	Cross sectional	Suspected ZIKV infections	DENV	18	38	47.7%	2c
Waggoner *et al*^13^ (2016)	Nicaragua	N/A	Sept 2015– Apr 2016	Cross sectional	Suspected arbovirus infections	CHIKV/ DENV	6	75	8.0%	2c
Carrillo-Hernández *et al*^9^ (2018)	Colombia	East	Aug 2015– Apr 2016	Cross sectional	Suspected arbovirus infections	CHIKV/ DENV	8	29	27.6%	2c

*Online supplementary table 2 gives details of all study populations in cohort studies and case reports.

†All articles were rated according to level of evidence using the Oxford Centre for Evidence-based Medicine's Levels of Evidence, March 2009.^11^

‡Cases originated from the National Surveillance System in Public Health from Colombia. Therefore, cases come from all over Colombia, with the condition of living in a place 2200 m above sea level.

CHIKV, chikungunya virus; DENV, dengue virus; N/A, not available; ZIKV, Zika virus.

**Table 2:** Summary of cohort and cross-sectional studies reporting on signs and symptoms of qRT-PCR-confirmed ZIKV coinfections (n=6 studies)

Author (year)	Location	Study year	Study design	Study population*	% female	Mean age (years)	N, total study population	Coinfecting agent(s)	N, coinfection cases	Other pathogens tested (negative)	Frequency of WHO ZIKV signs and symptoms (%)						Frequency of other reported signs and symptoms (%)		Level of evidence†
											Rash	Fever	Arthralgia	Conjunctivitis	Myalgia	Headache	URT symptoms	GI symptoms	
Mercado-Reyes *et al*^28^ (2018)	Colombia	2015/2016	Cross-sectional study	Suspected arbovirus infections	74	28	23 871	CHIKV	28	DENV	NR	NR	NR	NR	NR	NR	NR	NR	2c
Ball *et al*^20^ (2018)	Haiti	2014/2015	Cohort study	AFI cases	48	7.5	252	CHIKV	6	DENV2/ MAYV	0	33%	67%	NR	67%	17%	NR	17%	2b
Azeredo *et al*^19^ (2018)	Brazil	2016	Cross-sectional study	Suspected arbovirus infections	NR	34	134	DENV‡	18§	CHIKV	53%	73%	87%	60%	93%	67%	13%	40%¶	2c
Mercado-Reyes *et al*^28^ (2018)	Colombia	2015/2016	Cross-sectional study	Suspected arbovirus infections	74	28	23 871	DENV	3	CHIKV	NR	NR	NR	NR	NR	NR	NR	NR	2c
Araújo *et al*^38^ (2019)	Brazil	2014/2016	Cross-sectional study	AFI cases	NR	NR	9	DENV	1	CHIKV	100%	NR	100%	NR	NR	100%	NR	100%	2c
Alva-Urcia *et al*^30^ (2016)	Peru	2016	Cross-sectional study	AFI cases	63	NR	139	DENV	1	NR	NR	100%	NR	NR	NR	NR	NR	NR	2c
de Souza Costa *et al*^42^ (2019)	Brazil	2015/2016	Cross-sectional study	AFI cases	59	NR	453	MAYV	1	DENV, CHIKV, YFV, SLEV, ILHV, ROCV, WNV, EEEV, WEEV, VEEV	NR**	NR**	NR**	NR**	NR**	NR**	NR**	NR**	2c

URT symptoms: pharyngitis, sore throat, cough, pharyngeal congestion, adenopathy. GI symptoms: nausea, diarrhoea, vomiting, constipation, stomach ache.

*Online supplementary table 2 gives details of all study populations.

†All articles were rated according to level of evidence using the Oxford Centre for Evidence-based Medicine’s Levels of Evidence, March 2009.^11^

‡Seventeen cases of DENV-1 and one case of DENV-4.

§Signs and symptoms were only reported for 15 patients.

¶Forty per cent of cases presented with nausea, and 13% of cases with vomiting.

**Case was described to present with typical ZIKV signs for 3 days, but specific details were not reported.

AFI, acute febrile illness; CHIKV, chikungunya virus; DENV, dengue virus; EEV, east equine encephalitis virus; GI, gastrointestinal; ILHV, Ilheus virus; MAYV, Mayaro virus; NR, not reported; ROCV, Rocio virus; SLEV, Saint Louis encephalitis virus; URT, upper respiratory tract; VEEV, Venezuelan equine encephalitis virus; WEEV, west equine encephalitis virus; WNV, West Nile virus; YFV, yellow fever virus; ZIKV, Zika virus.

**Table 3:** Summary of case series studies reporting on signs and symptoms of different qRT-PCR-confirmed ZIKV coinfections (n=7 studies)

Author (year)	Location	Study year	Study design	Study population*	% female	Mean age (years)	N, total study population	Coinfecting agent(s)	N, coinfection cases	Other pathogens tested (negative)	Frequency of WHO ZIKV signs and symptoms (%)						Frequency of other reported signs and symptoms (%)		Level of evidence†
											Rash	Fever	Arthralgia	Conjunctivitis	Myalgia	Headache	URT symptoms	GI symptoms	
Acevedo *et al* (2017)^22^	Ecuador	2016	Case series	Cases with neurological symptoms‡	38	42	16	CHIKV	3	§	NR	100%	33%	NR	NR	33%	NR	NR	4
Metha *et al* (2018)^29^	Brazil	2015/2016	Case series	Cases with neurological symptoms§	50	52	22	CHIKV	2	DENV	100%	50%	50%	NR	NR	NR	NR	NR	4
Sardi *et al* (2016)^23^	Brazil	2015	Case series	AVI and of qRT-PCR ZIKV+ infections	NR	NR	15	CHIKV	2	DENV	50%	100%	50%	50%	100%	50%	NR	50%	4
Cabral-Castro *et al* (2016)^21^	Brazil	2015/2016	Case series	Suspected DENV infections	NR	NR	30	CHIKV	1	DENV	100%	100%	0	0	NR	NR	NR	100%	4
Estofolete *et al* (2018)^18^	Brazil	2016	Case series	Suspected arbovirus infections	42	46	1254	DENV	12	CHIKV	58%	58%	50%	25%	83%	75%	NR	17%	4
Chia *et al* (2017)^15^	Singapore	2016	Case series	Suspected ZIKV infections	NR	NR	163	DENV	4	NR	100%	100%	50%	50%	75%	50%	25%	75%	4
Li *et al* (2017)^31^	Singapore	2016	Case series	Suspected ZIKV infections	50	11	14	DENV	1	CHIKV	100%	100%	100%	100%	100%	100%	0	NR	4
Acevedo *et al* (2017)^22^	Ecuador	2015/2016	Case series	Cases with neurological symptoms‡	38	42	16	CHIKV, DENV	4	§	NR	50%	NR	NR	NR	25%	25%	25%	4
Acevedo *et al* (2017)^22^	Ecuador	2016	Case series	Cases with neurological symptoms‡	38	42	16	CHIKV, HIV, Toxo	1	§	NR	100%	NR	NR	NR	100%	NR	NR	4

URT symptoms: pharyngitis, sore throat, cough, pharyngeal congestion, adenopathy. GI symptoms: nausea, diarrhoea, vomiting, constipation, stomach ache.

*Online supplementary table 2 gives details of all study populations.

†All articles were rated according to level of evidence using the Oxford Centre for Evidence-based Medicine’s Levels of Evidence, March 2009^11^

‡Associated with suspected arbovirus infection.

§Tested for DENV; gram stain, HSV1/2/6, CMV, EBV, VZ, Toxo, MTB, enterovirus.

AVI, acute viral illness; CHIKV, chikungunya virus; CMV, cytomegalovirus; DENV, dengue virus; GI, gastrointestinal; HSV, herpes simplex virus; MT, Mycobacterium tuberculosis; NR, not reported; Toxo, Toxoplasma gondii; URT, upper respiratory tract; VZ, varicella zoster.

**Table 4:** Summary of case reports reporting on signs and symptoms of qRT-PCR-confirmed ZIKV coinfections (n=21 reports)

Author (year)	Location	Study year	Sex	Age (years)	Coinfecting agent(s)	Other pathogens tested (negative)	WHO ZIKV signs or symptoms						Other reported signs or symptoms		Additional information	Level of evidence*
							Rash	Fever	Arthralgia	Conjunctivitis	Myalgia	Headache	URT symptoms	GI symptoms		
Brito *et al*^24^ (2017)	Brazil	2016	M	74	CHIKV	DENV, HIV, CMV, HTLV, Schisto, HSVI/2 cystcercosis	NR	†	†	NR	NR	NR	NR	†	**Complications:** Meningoencephalitis associated with Guillain-Barré syndrome (EMG confirmed)	5
Silva *et al* (2018)	Brazil	2016	M	30	CHIKV	DENV, bacterial/fungal and MTB infections	NR	†	†	NR	†	NR	NR	NR	**Comorbidities:** Systemic lupus erythematosus **Complications:** Persistent fever for 5 weeks and renal dysfunction. 2 months postinfection severe arthralgia, requiring arthroplasty. 10 months postinfection acute deterioration in renal function, respiratory insufficiency and sepsis. **Outcome:** Death	5
Cherabuddi *et al*^26^ (2016)	Colombia	2016	F	40	CHIKV	DENV	†	†	†	†	NR	NR	NR	NR	Back pain, retro-ocular pain **Complications:** Persistent severe arthralgia after 2 months **Outcome:** Sequelae	5
Zambrano *et al*^27^ (2016)	Ecuador	2016	M	43	CHIKV	DENV	†	†	†	†	NR	NR	†	NR	**Outcome:** Full recovery	5
Zambrano *et al*^27^ (2016)	Ecuador	2016	F	43	CHIKV	NR	‡	†	‡	†	†	†	NR	NR	**Outcome:** Full recovery	5
Zambrano *et al*^27^ (2016)	Ecuador	2016	F	57	CHIKV	DENV	NR	†	NR	NR	NR	†	NR	NR	Lumbar back pain **Complications:** Guillain-Barré syndrome (EMG confirmed) **Outcome:** Full recovery	5
Azeredo *et al*^19^ (2018)	Brazil	2016	F	NR	DENV	CHIKV	†	†	NR	NR	NR	†	NR	†	Retro-orbital pain **Complications:** Suspected vertical transmission, pregnancy outcome alive newborn with functional plagiocephaly a flat spot on the back or side of newborn’s skull **Gestational age at infection:** 9 weeks	5
Azeredo *et al*^19^ (2018)	Brazil	2016	F	NR	DENV	CHIKV	†	†	NR	†	NR	NR	NR	NR	Retro-orbital pain **Complications:** Suspected vertical transmission to deceased newborn with respiratory insuf-ficiency **Gestational age at infection:** 20 weeks	5
Dupont-Rouzeyrol *et al*^32^ (2015)	New Caledonia	NR	F	38	DENV 1	NR	†	†	†	†	†	†	NR	†	Asthenia, retro-ocular pain **Outcome:** Full recovery	5
Dupont-Rouzeyrol *et al*^32^ (2015)	New Caledonia	NR	M	14	DENV 3	NR	NR	†	†	NR	†	†	NR	NR	Asthenia **Outcome:** Full recovery	5
Iovine *et al* 33 (2017)	United States§	2016	F	26	DENV 2	CHIKV	†	†	NR	†	NR	NR	†	†	Retro-orbital pain, fatigue, malaise, facial flushing **Outcome:** Full recovery	5
Villamil-Gomez *et al*^35^ (2016)	Colombia	NR	F	33	CHIKV, DENV	*Plasmodium* spp	†	NR	†	†	‡	†	NR	‡	Physical examination revealed cervical lymphadenopathy, bipalpebral oedema, and painful oedema in the lower limbs **Outcome:** Weekly obstetric ultrasounds from 14.6 to 29 weeks of gestation were normal	5
Gunturiz *et al*^36^ (2018)	Colombia	2016	F	18	CHIKV, Toxo	NR	NR	NR	NR	NR	NR	NR	NR	NR	**Complications:** Suspected vertical transmission of ZIKV infections to the fetus with outcome of fetus diagnosed with CZS at 20 weeks of gestation, termination at 29 weeks of gestation	5
Villamil-Gomez *et al*^34^ (2018)	Colombia	2015/2016	M	28	HIV	NR	†	†	NR	†	NR	NR	NR	NR	Recently diagnosed with HIV (<1 year), **Lymphocytes T CD4 count (cells/mm**^**3**^**):** 450, **HIV viral load (RNA copies/mL):** 100 **Complication:** Demyelination was found (EMG findings)	5
Villamil-Gomez *et al*^34^ (2018)	Colombia	2015/2016	F	49	HIV	NR	NR	NR	NR	NR	NR	NR	NR	†	Recently diagnosed with HIV (<1 year) **Lymphocytes T CD4 count (cells/mm**^**3**^**):** 98 **HIV viral load (RNA copies/mL):** 1800 Hypotension, dysarthria, decreased muscle strength, relaxation of sphincters, areflexia and basal bilateral crackles in the lungs **Complications:** Sepsis	5
Villamil-Gomez *et al*^34^ (2018)	Colombia	2015/2016	F	45	HIV	NR	†	†	NR	†	NR	NR	NR	†	Recently diagnosed with HIV (1 year ago) **Lymphocytes T CD4 count (cells/mm^3^):** 380 **HIV viral load (RNA copies/mL):** 800 **Complication:** Demyelination was found (EMG findings)	5
Valdespino- Vazquez *et al*^37^ (2018)	Mexico	2016	F	22	EBV, HHV6	DENV, CHIKV, WNV, VZ, HSV-I/2, HHV7, HHV8, CMV	†	†	NR	NR	NR	†	NR	NR	Infection of pregnant women at 14 weeks of gestation **Complications:** Suspected vertical transmission of ZIKV infections to the foetus with outcome of diagnosed CZS and fetal death at 30 weeks of gestational age, 4 hours after birth	5
Araujo *et al*^38^ (2018)	Brazil	2016	M	26	HSV-1	DENV, HSV, VZ, EBV, Toxo, HepC/B, Syphilis spp.	NR	†	NR	NR	NR	†	NR	†	**Complications:** Meningoencephalitis **Outcome:** Full recovery	5
Biron *et al* 39 (2016)	New Caledonia	NR	M	19	*Leptospira* spp	CHIKV, DENV	NR	†	NR	NR	†	NR	†	NR	**Complications:** Haemodynamic condition was unstable, sceptic shock **Outcome:** Full recovery	5
Neaterour *et al* (2017)	Puerto Rico	2016	M	48	*Leptospira* spp	DENV, CHIKV	NR	†	NR	NR	†	†	NR	†	**Complications:** Severe thrombocytopenia, persistent hypotension (BP=60/40), and onset of haematochezia suggestive of an acute gastrointestinal bleed. Haemodynamic instability and haemorrhagic manifestations. Cardiac arrest. **Outcome:** Death.	5
Alves *et al*^41^ (2017)	Brazil	NR	M	NR, a boy	*Schistosoma mansoni*	NR	†	†	NR	NR	NR	NR	NR	NR	**Complications:** Inflammation of the right testicle and epididymis. The microscopic examination of the testis ruled out the possibility of cancer but confirmed the diagnosis of extensive loss of testicular structure and schistosome egg-induced granulomas. **Outcome:** Full recovery	5

URT symptoms: pharyngitis, sore throat, cough, pharyngeal congestion, adenopathy. GI symptoms: nausea, diarrhoea, vomiting, constipation, stomach ache.

*All articles were rated according to level of evidence using the Oxford Centre for Evidence-based Medicine’s Levels of Evidence, March 2009.^11^

†Reported to be present.

‡Reported not to be present.

§Travel associated diagnosed from Haiti.

CHIKV, chikungunya virus; CMV, cytomegalovirus; CZS, congenital Zika syndrome; DENV, dengue virus; EMG, electromyography testing; GI, gastrointestinal; Hep, hepatitis virus; HHV, human herpes virus; HSV, herpes simplex virus; HTLV, human T-lymphotropic virus; MTB, Mycobacterium tuberculosis; NR, not reported; Toxo, Toxoplasma gondii; URT, upper respiratory tract; VZ, varicella zoster; WNV, West Nile virus.

### ZIKV coinfection types

**Figure 3:**
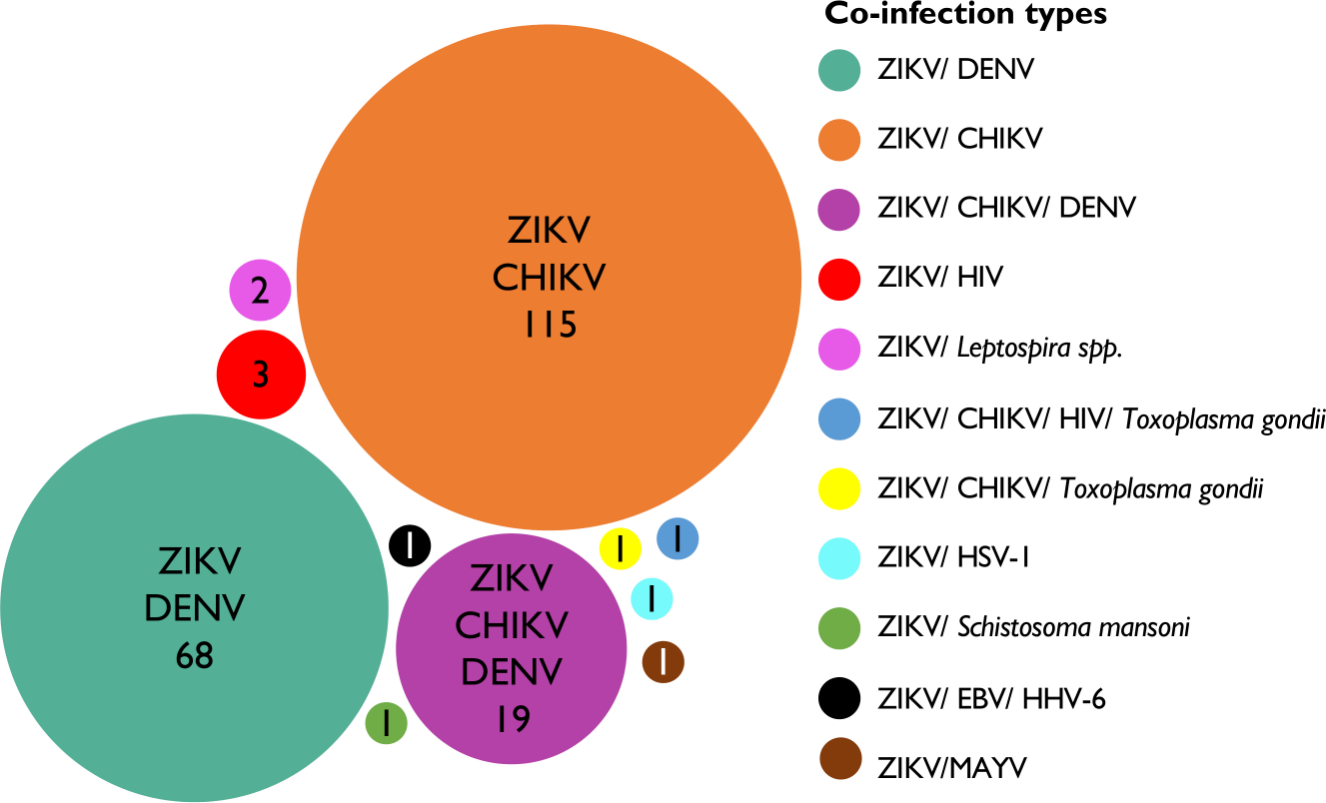
Zika virus coinfection types identified in this systematic review. Size of circles represents the number of cases reported per coinfection type. In total, 213 coinfection cases were included, ie, ZIKV/CHIKV (n=115), ZIKV/DENV (n=68), ZIKV/CHIKV/DENV (n=19), ZIKV/HIV (n=3), ZIKV/*Leptospira* spp (n=2), ZIKV/HIV/*Toxoplasma gondii* (n=1), ZIKV/CHIKV/*Toxoplasma gondii* (n=1), ZIKV/HSV-1 (n=1), ZIKV/*Schistosoma mansoni* (n=1), ZIKV/EBV/HHV-6 (n=1), ZIKV/MAYV (n=1. CHIKV, chikungunya virus; DENV, dengue virus; EBV, Epstein-Barr virus; HHV, human herpes virus; HSV, herpes simplex virus; MAYV, Mayaro virus; ZIKV, Zika virus.

ZIKV infections were most frequently reported to occur concurrently with other arthropod-borne viruses (arboviruses). Out of the 213 coinfections examined, there were 115 ZIKV/chikungunya virus (CHIKV) coinfection cases, 68 ZIKV/dengue virus (DENV) coinfection cases and 19 cases coinfected with all three viruses. Other reported ZIKV coinfections included ZIKV/HIV (n=3), ZIKV/*Leptospira* spp (n=2), ZIKV/CHIKV/HIV/*Toxoplasma gondii* (n=1), ZIKV/CHIKV/*Toxoplasma gondii* (n=1), ZIKV/Epstein-Barr virus (EBV)/human herpes viruses-6 (HHV-6) (n=1), ZIKV*/*herpes simplex virus-1 (HSV-1) (n=1), ZIKV*/*Mayaro virus (MAYV) (n=1) and ZIKV*/Schistosoma mansoni* (n=1) (figure 3).

### Frequencies of ZIKV coinfections

The frequencies of coinfections among ZIKV-infected populations were reported in 11 studies, including 1 cohort study, 7 cross-sectional studies and 3 case series (table 1, online supplementary table 2). Frequency estimates were reported only for coinfections with CHIKV and DENV and varied geographically and across study populations at risk. Among patients presenting with arbovirus-like symptoms, ZIKV/CHIKV coinfection frequencies were reported to range from 0.3% in a study in Colombia to 54% in a study in Brazil.^5 9 12–14^ Similarly, ZIKV/DENV coinfection frequencies in patients with arbovirus-like symptoms were reported to range from 0.03% in a study in Colombia to 47.4% in a study in Brazil.^9 13 15–19^ ZIKV/CHIKV/DENV coinfection frequencies ranged from 8% in a study in Nicaragua to 27.6% in a study in Colombia.^9 13^

### Signs and symptoms of coinfections

In total, 27 studies, including 1 cohort study, 5 cross-sectional studies, 7 case series and 14 case report studies, reported the signs and symptoms of ZIKV coinfection across a total of 106 ZIKV-coinfected cases.

### ZIKV/CHIKV coinfections

The clinical presentations of 48 cases with ZIKV/CHIKV coinfection were reported in 1 cohort study, 1 cross-sectional study, 4 case series and 6 case reports (tables 2–4, online supplementary tables 1 and 2).^20–28^ Within the cohort, cross-sectional and case series studies, cases were reported to present with the following signs and symptoms consistent with the WHO ZIKV clinical case definition^1^: fever (33%–100%), rash (0%–100%), conjunctivitis (0%–50%), myalgia (67%–100%), arthralgia (0%–67%) and headache (17%–50%) (tables 2 and 3). In addition, gastrointestinal (GI) symptoms were reported in 17% to 100% of cases in three studies (tables 2 and 3).^20 21 23^

**Figure 4:**
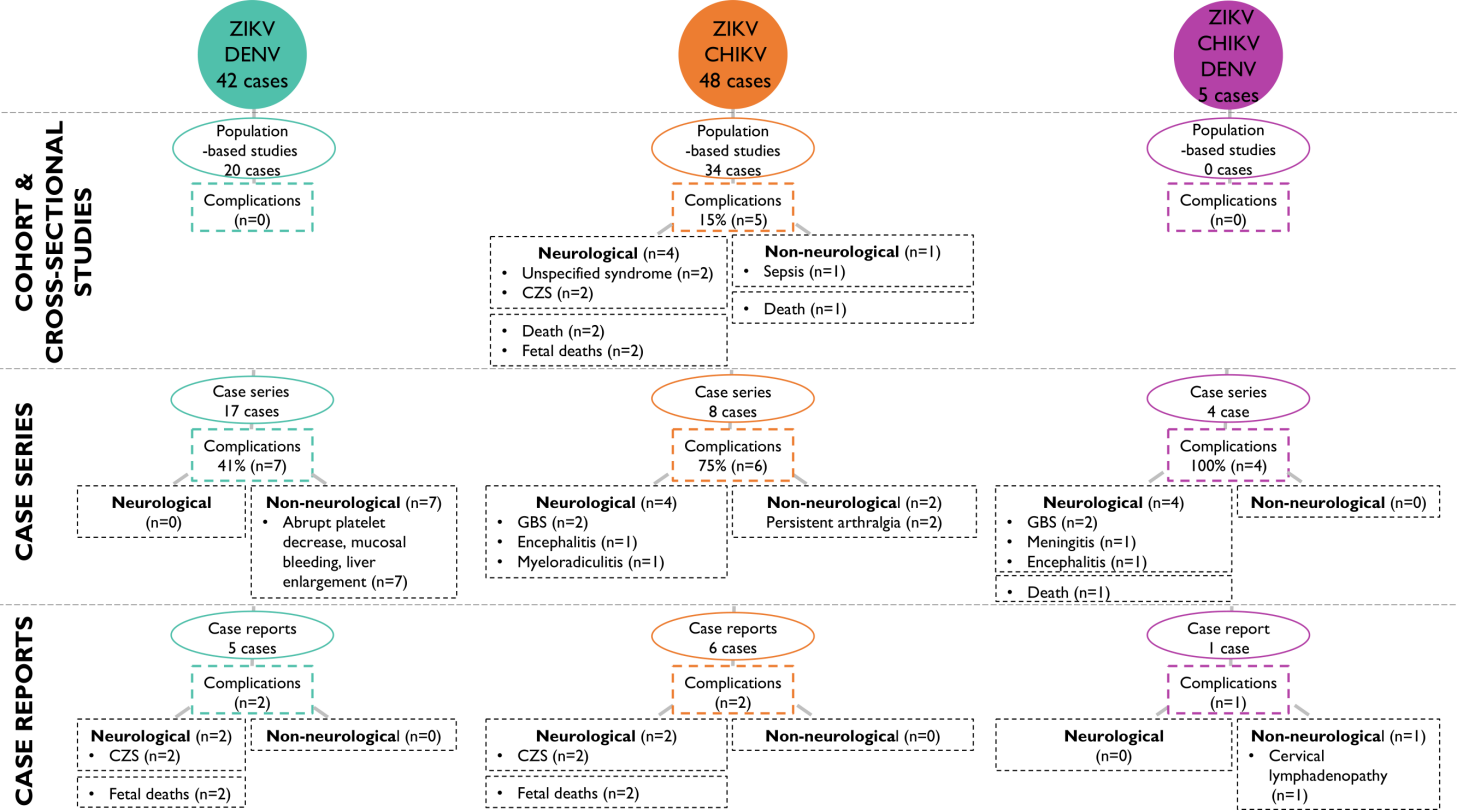
Complications resulting from Zika virus coinfections with CHIKV and DENV by study design. In cohort and cross-sectional studies, 15% of ZIKV/CHIKV coinfections resulted in complications. In case series, 41% of ZIKV/DENV, 75% of ZIKV/CHIKV and 100% of ZIKV/CHIKV/DENV cases resulted in in complications. In case reports, two ZIKV/DENV, two ZIKV/CHIKV and one ZIKV/CHIKV/DENV coinfections resulted in complications. CHIKV, chikungunya virus; CZS, congenital Zika syndrome; DENV, dengue virus; GBS, Guillain-Barré syndrome; n, number of complications; ZIKV, Zika virus.

Complications were reported among 14.7% (5 cases) of ZIKV/CHIKV-coinfected cases in cohort and cross-sectional studies,^22–27^ of whom two adult cases presented with unspecified neurological complications that resulted in death (figure 4).^28^ Additionally, two coinfections in pregnancy were associated respectively with anencephaly and an absence of a heartbeat.^28^ A non-neurological complication reported was a case that died from multiorgan failure following haemorrhagic manifestations.^23 28^ The case series studies described that six out of eight ZIKV/CHIKV-coinfected cases developed complications, which included neurological manifestations, such as GBS in two cases,^22 29^ encephalitis in one case,^22^ myeloradiculitis in one case,^29^ as well as non-neurological complications, such as persistent severe arthralgia in one case.^23^ Additionally, four case reports described ZIKV/CHIKV coinfection-associated complications, including GBS in two cases,^24 27^ persistent severe arthralgia in one case^26^ and sepsis resulting in death in one case.^25^

### ZIKV/DENV coinfections

The clinical features of 42 cases with ZIKV/DENV coinfection were described across four cross-sectional studies, three case series and five case reports (tables 2–4, online supplementary tables 1 and 2).^15 18 19 30–33^ Cases with ZIKV/DENV coinfection within the cross-sectional and case series studies were reported to present with the following signs and symptoms consistent with the WHO ZIKV clinical case definition^1^: fever (58%–100%), rash (53%–100%), conjunctivitis (25%–100%), myalgia (75%–100%), arthralgia (50%–100%) and headache (50%–100%) (tables 2 and 3). Other reported clinical features included GI symptoms in 17%–75% of cases and upper respiratory tract (URT) symptoms in 13%–25% of cases (tables 2 and 3).

Complications were reported among none of the ZIKV/DENV-coinfected individuals in cohort and cross-sectional studies (figure 4). However, seven cases with complications were reported in case series, which presented respectively with painful hepatomegaly, liver enlargement, mucosal bleeding, gingival bleeding, significant thrombocytopenia and abrupt platelet decrease.^15 18 19^ The only neurological complications resulting from ZIKV/DENV coinfection were reported in two case reports documenting infections in pregnancy, with one case resulting in a newborn with functional plagiocephaly and the other in fetal death (table 3).^19^

### ZIKV/CHIKV/DENV coinfections

The clinical presentation of five cases with ZIKV/CHIKV/DENV coinfection were described in one case series (four cases) and one case report (tables 3 and 4, online supplementary tables 1 and 2).^22 34^ Similar to ZIKV/CHIKV and ZIKV/DENV-coinfected cases, ZIKV/CHIKV/DENV-coinfected cases presented with signs and symptoms consistent with the ZIKV WHO clinical case definition.^1^ All five cases were reported to have complications (figure 4). The case series reported GBS in two cases, one case of meningitis and one case of encephalitis, which resulted in death. Notably, the study’s population was selected to include only clinical patients presenting to hospital with neurological symptoms.^22^ The case report documented one case of cervical lymphadenopathy in pregnancy and full recovery.^35^

### Other ZIKV coinfections

There is limited published evidence on ZIKV coinfections with other pathogens. To date, the clinical signs and symptoms of 10 cases with eight other ZIKV coinfection types have been documented in one cross-sectional study, one case series and seven case reports (tables 2–4, online supplementary tables 1 and 2).^34 36–42^

In addition to presenting with signs and symptoms consistent with the WHO ZIKV clinical case definition, almost all cases of ZIKV coinfections with pathogens other than DENV or CHIKV were reported to experience complications. Neurological complications were reported in two ZIKV/HIV coinfections, one ZIKV/CHIKV/HIV/*Toxoplasma gondii* coinfection and one ZIKV/HSV-1 coinfection. These neurological complications included meningitis, meningoencephalitis and demyelinations confirmed by electromyography.^22 34 38^ Further, one ZIKV/HIV-coinfected case developed sepsis, resulting in death.^34^ Two ZIKV/*Leptospira* spp-coinfected cases developed haemodynamic instability, one resulting in septic shock, and one in death.^39 40^ Additionally, one ZIKV/*Schistosma mansoni*-coinfected case experienced testicular inflammation with granulomas induced by schistosome eggs.^41^

Coinfections in pregnancy were described in three ZIKV coinfection types: ZIKV/MAYV, ZIKV/CHIKV/*Toxoplasma gondii* and ZIKV/EBV/HHV6 coinfections.^36 37 42^ In the latter two, vertical ZIKV transmission was suspected, as both fetuses were diagnosed with CZS. After diagnosis, one pregnancy was terminated at 29 weeks of gestation and one newborn died 4 hours after birth at 30 weeks of gestation due to respiratory distress syndrome.

### Levels of evidence

The levels of evidence for the studies were assessed using the OCEBM Levels of Evidence (1=highest, 5=lowest). Two cohort studies with limited follow-up were graded evidence level 2b.^5 20^ Ten cross-sectional studies were graded evidence level 2c.^9 12–14 17 19 28 30 42 43^ Eight case series studies were graded evidence level 4.^15 16 18 21–23 29 31^ Fourteen case report studies were graded evidence level 5.^19 24–27 32–41^ Thus, most of the studies included in the systematic review are evidence level 4 or 5.

## Discussion

This systematic review summarises the existing literature on ZIKV coinfections. Specifically, it describes the estimated frequencies of reported ZIKV coinfections and their clinical spectrum. The search identified 34 studies conducted between 2014 and 2019, which reported 213 cases of ZIKV coinfection with 10 different pathogens. ZIKV coinfections were detected across 10 countries, primarily in Latin America. CHIKV and DENV were the predominantly reported ZIKV coinfecting agents and the only ZIKV coinfections for which population frequencies were described. ZIKV coinfection frequencies among ZIKV-infected cases varied significantly between location and population type. The vast majority of ZIKV-coinfected cases were reported to present with the signs and symptoms described for uncomplicated ZIKV monoinfections and defined by the WHO.^1^ However, complications were reported to arise in 9% of ZIKV-coinfected cases in cohort and cross-sectional studies.

This is the first systematic review to study how frequently individuals with ZIKV infection have a coexisting infection of any kind. The variation in frequencies reported for ZIKV/arbovirus coinfections among the ZIKV-infected individuals reported in this study was likely influenced by differences in study design and the selected study population. Factors, such as study location, season and study period in relation to the ZIKV outbreak, will have additionally influenced ZIKV coinfection frequency estimates. As expected, ZIKV coinfections were relatively more common in studies conducted during concurrent arbovirus outbreaks.^14 44^ These differences in study design, timing and location make it difficult to generalise ZIKV coinfection frequency estimates, but provide important knowledge that arbovirus coinfections can occur in up to half of ZIKV-infected cases in certain contexts. Our findings are consistent with a systematic review of CHIKV/DENV coinfections, which found the frequency of CHIKV/DENV coinfections reported in 28 studies ranged from 1% to 36%.^45^ The heterogeneity across studies also reflects the difficulty in estimating the background level of ZIKV infections (ie, the denominator for assessing coinfection frequencies), given the diagnostic challenges in identifying acute ZIKV infections.^46^

Overall, the evidence identified in this review suggests that ZIKV coinfections appear to present with a mild clinical presentation similar to that previously described for ZIKV monoinfections. Of note, GI and URT symptoms, which are considered uncharacteristic for ZIKV, were reported to occur not infrequently in ZIKV/DENV, ZIKV/CHIKV and ZIKV/CHIKV/DENV-coinfected cases. While the evidence base from animal model studies of ZIKV coinfection is limited to date, two studies have compared ZIKV infection among rhesus macaque models with and without simian immunodeficiency virus or chimeric simian HIV.^47 48^ Whereas coinfected macaques were observed to have lower peak Zika viral loads with a longer clearance time in both investigations, the area under the viral load curves did not appear to differ substantively by coinfection status, potentially suggesting an overall limited impact of coinfection on disease progression but raising questions about the role of lentiviral coinfection in onward transmission.^47 48^

Although the existing reports suggest that coinfections do not appear to markedly alter the clinical presentation of uncomplicated ZIKV disease in humans, the findings from this review highlight a need for additional high quality research investigating whether coinfections may influence complication risks. Based on the limited available evidence, the complications described for ZIKV coinfections appear to be broadly similar to those reported for ZIKV monoinfections.^49^ However, 33% of the coinfection-related complications appeared to be atypical for ZIKV monoinfections, but were consistent with complications previously documented for the coinfecting pathogens (eg, bleeding in 10% of ZIKV/DENV cases and persistent arthralgia in 6% of ZIKV/CHIKV cases).^50 51^ In addition, among deaths of ZIKV-coinfected cases, three of the nine cases had immune deficiencies and one ZIKV/*Leptospira* spp-coinfected case died from complications established for *Leptospira* spp infections.^40^ The remaining five deaths reported from ZIKV coinfections were three fetal deaths, one case following multiorgan failure and one case following encephalitis.^22 28^ Additionally, some complications may have been missed, especially those that occurred after the acute infections, as the follow-up period of the individual studies may have not been adequate to detect late-onset complications. Further research (eg, an ongoing cohort study of ZIKV/HIV coinfections in pregnant women^52^) will be valuable for discerning the relative risk of complications of ZIKV coinfection versus monoinfections.

This review had strengths and limitations. ZIKV is an emerging infectious disease of significant public health concern, and this is the first systematic review of the frequency, types and clinical presentation of ZIKV coinfections. The study employed a broad search strategy including search terms for all potential coinfecting pathogens and using multiple languages to identify all available evidence. Most importantly, the review included only qRT-PCR-confirmed ZIKV coinfections, which is the most accurate way to diagnose acute coinfections (ie, due to the very short time window of qRT-PCR testing (<7 days)) and limits misdiagnosis, which is of particular importance with the high cross-reactivity reported from arbovirus serology testing. On the other hand, by focusing on concurrent infections, the current review was unable to appraise the potential impact of recent infections; for example, it has been previously reported that pre-existing immunity to DENV, which shares a common vector and circulates in most of the countries reporting ZIKV coinfection, may influence the clinical presentation of ZIKV infection.^53^ The additional limitations of this review mainly stem from the lack of available high-quality evidence on ZIKV coinfections. Notably, the majority of included studies were rated level 4 or 5 according to the OCEBM Levels of Evidence. Only seven studies were rated level 2 or above. Additionally, the reported ZIKV coinfection types may have been influenced by the underlying prevalence of coinfecting pathogens in the population and the applied diagnostic practices (ie, multiplex testing vs testing on clinician's suspicion). The use of specific case definitions in included cross-sectional and case series studies (eg, fever and rash^15^) may have also introduced a selection bias that potentially led to an over-representation of specific symptoms associated with ZIKV coinfection reported for a given study (eg, reporting 100% of cases as presenting with fever and rash).^15^ Finally, the studies selected for this systematic review only included symptomatic ZIKV-infected cases, which represent only approximately 40% of all ZIKV cases.^2^ It is likely that the actual frequency of ZIKV coinfections may be higher as many cases will be asymptomatic and therefore never seek medical attention. However, the recently implemented multiplex PCR assay, which tests for CHIKV, DENV and ZIKV simultaneously, will likely improve the detection of ZIKV/arbovirus coinfections and facilitate future assessment of the frequency of ZIKV coinfections.^54^

In conclusion, the findings of this review suggest that the cocirculating arboviruses, CHIKV and DENV, are the most common ZIKV coinfection types and may, in specific populations and epidemiological contexts, occur in up to half of ZIKV infections. The evidence collated in this systematic review suggests coinfections do not markedly alter the generally mild clinical presentation of uncomplicated ZIKV disease. However, additional and better quality evidence should be prioritised in future outbreaks to corroborate the estimates of the frequency of ZIKV coinfections and to interrogate the importance of ZIKV coinfections in the development of ZIKV-related complications, especially for ZIKV coinfections with CHIKV and DENV.
