# Supplementary material for: The frequency and clinical presentation of Zika virus coinfections: a systematic review
Source: BMJ Glob Health. 2020 May 7;5(5):e002350. doi: 10.1136/bmjgh-2020-002350 (PMC7228501; doi:10.1136/bmjgh-2020-002350)
Supplement: Supplementary data [file bmjgh-2020-002350supp001.pdf]

SUPPLEMENTARY APPENDIX

Supplementary Table 1 ***Detail of cases in cross-sectional studies and case series (Table 2 & 3) reporting on signs and symptoms of qRT-PCR-confirmed ZIKV co-infections.***

Author (year)	Location	Study year	Sex	Age (y)	Co–infecting agent	Other pathogens tested (negative)	WHO ZIKV signs or symptoms						Other reported signs or symptoms		Additional information
							Rash	Fever	Arthralgia	Conjunctivitis	Myalgia	Headache	URT symptoms	GI symptoms	
**Acevedo *et al.* (2017)**	Ecuador	2016	F	54	**CHIKV**	DENV; Gram-stain, HSV-1/2/6, CMV, EBV, VZ, Toxo, MTB, enterovirus [a]	NR	■	NR	NR	NR	■	NR	NR	Lumbar pain **Complications:** Guillain-Barré Syndrome (EMG confirmed)[c]
**Acevedo *et al.*(2017)**	Ecuador	2016	M	44	**CHIKV**	[a]	NR	■	■	NR	NR	NR	NR	NR	NR
**Acevedo *et al.*(2017)**	Ecuador	2016	M	47	**CHIKV**	[a]	NR	■	NR	NR	NR	NR	NR	NR	Sleep disturbance **Complications:** Tremors and walking difficulties. Tonic-clonic seizure, Encephalitis
**Cabral-Castro *et al.*(2016)**	Brazil	2015/16	F	28	**CHIKV**	DENV	■	■	☐	☐	NR	NR	NR	■	Retro-ocular pain **Complications:** Post infection relapse with pruritic rash no fever
**Mercado-Reyes *et al.* (2018)[b]**	Colombia	2015/16	NR	47	**CHIKV**	DENV	☐	☐	☐	NR	NR	NR	NR	NR	**Comorbidities:** Hypertension, diabetes mellitus, obesity

1

															**Complications:** Neurological syndrome<br>**Outcome:** Death
**Mercado-Reyes et *al.* (2018)** [b]	Colombia	2015/16	NR	78	**CHIKV**	DENV	□	□	□	NR	NR	NR	NR	NR	**Comorbidities:** Hypertension, diabetes mellitus, chronic kidney disease, chronic cardiac disease<br>**Complications:** Neurological syndrome<br>**Outcome:** Death
**Mercado-Reyes et *al.* (2018)** [b]	Colombia	2015/16	NR	28	**CHIKV**	DENV	□	■	■	NR	NR	NR	NR	NR	**Complications:** Haemorrhagic manifestations, Multi organ failure<br>**Outcome:** Death
**Mercado-Reyes et *al.* (2018)** [b]	Colombia	2015/16	F	22	**CHIKV**	DENV	NR	NR	NR	NR	NR	NR	NR	NR	Infection in pregnancy<br>**Complications :** Intrauterine growth restriction, ancephaly<br>**Outcome:** Fetal death
**Mercado-Reyes et *al.* (2018)** [b]	Colombia	2015/16	F	22	**CHIKV**	DENV	NR	NR	NR	NR	NR	NR	NR	NR	Infection in pregnancy<br>**Complications :** absence of heart beat<br>**Outcome:** Fetal death
**Metha et *al.* (2018)**	Brazil	2015/16	M	34	**CHIKV**	DENV	■	NR	NR	NR	NR	NR	NR	NR	**Complications:** Guillain-Barré Syndrome (EMG confirmed)[c]<br>**Outcome:** Full recovery
**Metha et *al.* (2018)**	Brazil	2015/16	M	65	**CHIKV**	DENV	■	■	■	NR	NR	NR	NR	NR	**Complications:** Myeloradiculitis<br>**Outcome:** Full recovery
**Sardi et *al.* (2016)**	Brazil	2015	F	48	**CHIKV**	DENV	□	■	■	□	■	■	NR	■	**Complications:** Severe arthralgia<br>**Outcome**: Sequelae

**Sardi *et al.* (2016)**	Brazil	2015	F	40	**CHIKV**	DENV	■	■	NR	■	■	NR	NR	NR	Painful posterior cervical lymph node measuring 5 mm **Outcome:** Full recovery
**Alva-Urcia *et al.*(2016)**	Peru	2016	NR	NR	**DENV**	NR	NR	■	NR	NR	NR	NR	NR	NR	NR
**Silva *et al.* (2019)**	Brazil	2014/ 16	NR	NR	**DENV**	CHIKV	■	NR	■	NR	NR	■	NR	■	Retro-orbital pain
**Estofolete *et al.*(2018)**	Brazil	2016	F	63	**DENV 1**	CHIKV	■	■	■	NR	■	■	NR	NR	NR
**Estofolete *et al.*(2018)**	Brazil	2016	M	27	**DENV 1**	CHIKV	■	■	NR	NR	■	NR	NR	NR	NR
**Estofolete *et al.*(2018)**	Brazil	2016	F	64	**DENV 1**	CHIKV	NR	NR	NR	NR	NR	NR	NR	■	NR
**Estofolete *et al.*(2018)**	Brazil	2016	M	68	**DENV 1**	CHIKV	NR	■	■	NR	■	■	NR	NR	**Complications :** Abrupt platelets decrease
**Estofolete *et al.*(2018)**	Brazil	2016	M	33	**DENV 1**	CHIKV	NR	■	NR	NR	■	■	NR	■	**Complications :** Abrupt platelets decrease
**Estofolete *et al.*(2018)**	Brazil	2016	M	62	**DENV 1**	CHIKV	NR	■	NR	NR	■	■	NR	NR	NR
**Estofolete *et al.*(2018)**	Brazil	2016	F	36	**DENV 1**	CHIKV	■	■	NR	NR	■	■	NR	NR	NR
**Estofolete *et al.*(2018)**	Brazil	2016	M	25	**DENV 2**	CHIKV	■	■	NR	■	■	■	NR	■	NR
**Estofolete *et al.*(2018)**	Brazil	2016	F	39	**DENV 2**	CHIKV	■	■	■	NR	NR	NR	NR	NR	NR
**Estofolete *et al.*(2018)**	Brazil	2016	M	48	**DENV 2**	CHIKV	■	■	■	■	■	■	NR	NR	NR

**Estofolete *et al.*(2018)**	Brazil	2016	F	47	**DENV 2**	CHIKV	■	NR	■	■	■	■	NR	■	NR
**Estofolete *et al.*(2018)**	Brazil	2016	M	41	**DENV 2**	CHIKV	NR	NR	■	NR	■	■	NR	■	NR
**Chia *et al.* (2017)**	Singa-pore	2016	M	42	**DENV 3**	NR	■	■	NR	■	■	■	NR	■	NR
**Chia *et al.* (2017)**	Singa-pore	2016	F	45	**DENV 1**	NR	■	■	NR	NR	■	NR	NR	■	**Complications :** Malaise, gingival bleeding
**Chia *et al.* (2017)**	Singa-pore	2016	M	40	**DENV 1**	NR	■	■	■	■	■	NR	NR	■	NR
**Chia *et al.* (2017)**	Singa-pore	2016	M	15	**DENV**	NR	■	■	■	NR	NR	■	■	NR	**Complications :** Developed significant thrombocytopenia
**Li *et al.* (2017)**	Sing-apore	2016	M	15	**DENV**	CHIKV	■	■	■	■	■	■	☐	NR	Bilateral knee pain<br>**Outcome:** Full recovery
**Acevedo *et al.*(2017)**	Ecuador	2016	M	18	**CHIKV, DENV**	[a]	NR	NR	NR	NR	NR	NR	■	NR	**Complications:** Guillain-Barré Syndrome (EMG confirmed)[c]
**Acevedo *et al.*(2017)**	Ecuador	2016	M	23	**CHIKV, DENV**	[a]	NR	NR	NR	NR	NR	NR	NR	NR	**Complications:** Encephalitis<br>**Outcome:** Death
**Acevedo *et al.*(2017)**	Ecuador	2016	F	25	**CHIKV, DENV**	[a]	NR	■	NR	NR	NR	■	NR	■	Followed by generalized tonic-clonic seizure, ptosis of the eyelids, neck stiffness<br>**Complications**: Meningitis
**Acevedo *et al.*(2017)**	Ecuador	2016	M	62	**CHIKV, DENV**	[a]	NR	■	NR	NR	NR	NR	NR	NR	Sweating, paraplegia, arreflexia, dyspnea, decreased muscular strength in arms<br>**Complications** : Guillain-Barré Syndrome (EMG confirmed)[c]

**Acevedo et *al.*(2017)**	Ecuador	2016	F	28	**CHIKV HIV, Toxo**	a	NR	■	NR	NR	NR	■	NR	NR	**Complications:** Meningitis
**Souza Costa *et al.* *(2019)***	Brazil	2015/16	F	35	**MAYV**	DENV, YFV, SLEV, ILHV, ROCV, WNV, EEEV,WEEV, VEEV	NR	NR	NR	NR	NR	NR	NR	NR	Infection in third trimester of pregnancy **Outcome:** Newborn presented without any congenital abnormalities

■ *Reported to be present;* ☐ *Reported not to be present; Not reported (**NR**); Upper Respiratory Tract (**URT**) symptoms: pharyngitis, sore throat, cough, pharyngeal congestion, adenopathy; Gastro-intestinal (**GI**) symptoms: nausea, diarrhea, vomiting, constipation, stomach ache; Zika virus (**ZIKV**); Chikungunya virus (**CHIKV**); Dengue virus (**DENV**); Herpes simplex virus (**HSV**); Cytomegalovirus (**CMV**); Varizella zoster (**VZ**); Toxoplasma gondii (**Toxo**); Mycobacterium tuberculosis (**MTB**); Mayaro virus (**MAYV**); Yellow fever virus (**YFV**); West Nile virus (**WNV**); Saint Louis Encephalitis virus (**SLEV**); Rocio virus (**ROCV**); Ilheus virus (**ILHV**); East, West, Venezuelan equine encephalitis virus (**EEV,WEEV,VEEV**), Electromyography testing (**EMG**);* [a] *tested for* DENV; Gram-stain; HSV-1/2/6; CMV; EBV; VZ; Toxo; MTB; enterovirus. [b] *Details were only reported on five fatal ZIKV/CHIKV cases.* [c] *Electromyography testing (**EMG**).*

1

5

Supplementary Table 2 ***Details of study populations in cohort studies, cross-sectional studies, and case series (n = 20 studies).***

**Author (year)**	**Study population**	**Details of study population as described in publication**
**Acevedo *et al.* (2017)**	Cases with neurological symptoms and suspected arbovirus infections	Cases with neurological symptoms and/ or concern for acute arboviral infections without case definition.
**Alva-Urcia *et al.* (2016)**	AFI cases	Cases who arrived to Internal Medicine-Pediatrics outpatient clinics with AFI (greater than or equal to 38°C axillary temperature in the previous 7 days) along with one or more of the following symptoms: headache, muscle pain, retro-ocular pain, joint pain, nausea, low appetite, vomiting, dizziness, abdominal pain, diarrhea, chills, rash, photophobia, sore throat, cough, pallor, rhinorrhea, dyspnea, jaundice, cough, conjunctival injection, dysuria or convulsions; no cases with an identifiable source of infection, such as sinusitis, pneumonia, acute otitis media and acute upper respiratory tract infections, among others.
**Azeredo *et al.* (2018)**	Suspected arbovirus infections	Cases with fever and rash during acute phase of infection (up to the 7th day after disease onset) followed by at least two of the following signals and symptoms: headache, myalgia or arthralgia, conjunctivitis, pruritus, retro-orbital pain and prostration were recruited as suspicion of arboviral infection.
**Ball *et al.* (2018)**	AFI cases	Cohort of school children of acute undifferentiated febrile illness (AFI).AFI defined as cases with fever and/or fever on presentation in a child with no obvious source of infection (ie, no respiratory symptoms, symptoms of urinary tract infection, or diagnostic criteria for malaria or typhoid).
**Brasil *et al.* (2016)**	Pregnant women with rash	Pregnant women with a rash that had developed in the previous 5 days.
**Cabral-Castro *et al.* (2016)**	Suspected DENV infections	Suspected DENV cases without case definition.
**Carrillo-Hernandez *et al.* (2018)**	Suspected arbovirus infections	Cases with febrile syndrome compatible with ZIKV, CHIKV and DENV infection, and in the acute phase of the disease, i.e., fever for no more than seven days.
**Charlys da Costa *et al.* (2017)**	Suspected arbovirus infections with rash	Cases with exanthemous illness symptoms compatible with ZIKV, CHIKV and DENV infection.
**Chia *et al.* (2017)**	Suspected ZIKV infections	Suspected ZIKV cases with fever, maculopapular rash, and any of the following: arthralgia, myalgia, headache, or non-purulent conjunctivitis.

**Souza Costa *et al.* *(2019)***	AFI cases	Cases notified as suspected ZIKV, CHIKV and DENV cases with National Surveillance System of Public Health (SINAN) of Brazil.
**Colombo *et al.* (2017)**	Suspected ZIKV infections	Cases with macular or papular rash and two or more of the following signs and symptoms: fever or conjunctival hyperemia without secretion and pruritus, polyarthralgia or joint edema
**Estofolete *et al.* (2018)**	Suspected arbovirus infections	Suspected DENV cases: fever, abdominal pain, vomiting, bleeding of the mouth, hemorrhage, volume of urine, breathing difficulties, feeling cold, and suspected Zika cases presence of macular or papular rash with two or more of following signs and symptoms: fever or conjunctival hyperemia without secretion.
**Li *et al.* (2017)**	Suspected ZIKV infections	Suspected ZIKV cases with fever, maculopapular rash, and any of the following: arthralgia, myalgia, headache, and non-purulent conjunctivitis.
**Magalhaes *et al.* (2017)**	Suspected arbovirus infections	Cases had to be older the age of 5 years and have fever or history of fever for less than 72 h, clinical symptoms were supposed to be consistent with possible dengue, i.e., suspicion of dengue and/or undifferentiated fever in a patient from a dengue endemic area however with no signs of severe disease. Cases were not included if there were localized features suggesting an alternative diagnosis, e.g., pneumonia, otitis, etc.
**Mercado-Reyes *et al.* (2018)**	Suspected arbovirus infections	Cases with suspected ZIKV, CHIKV and DENV infections, which was reported to the National Surveillance System of Public Health (SIVIGILA) of Colombia.
**Metha *et al.* (2018)**	Cases with neurological symptoms and suspected ZIKV infections	Cases with an acute neurological condition associated with a suspected ZIKV infection, as identified by fever, arthralgia or rash illness in the preceding three months.
**Pessoa *et al.* (2016)**	Suspected arbovirus infections	Cases with rash, conjunctivitis, and/or joint pain with or without fever or headache were examined further.
**Sardi *et al.* (2016)**	AVI and of qRT-PCR ZIKV+ infections	Acute viral illness without case definition, and positive ZIKV qRT-PCR result.
**Silva *et al.* (2019)**	Suspected arbovirus infections	Cases with suspected ZIKV, CHIKV and DENV infections .
**Waggoner *et al.* (2016)**	Suspected arbovirus infections	Cases with suspected ZIKV, CHIKV, and/or DENV infections.

*Acute Febrile Illness (**AFI**); Acute Viral illlness (**AVI**); Zika virus (**ZIKV**); Chikungunya virus (**CHIKV**); Dengue virus (**DENV**);*

*Supplementary Appendix 1***. *Literature Search Strategy: Search terms for each database***

Pubmed:

(zikv OR zika OR "Zika Virus"[Mesh] OR "Zika Virus Infection"[Mesh]) AND (coinfec* OR (co infec*) OR co-infec* OR “Coinfection”[Mesh] OR "Bacterial Infections"[Mesh] OR bacterial* OR bacteria* OR bacteri* OR bactéri* OR "Parasitic Diseases"[Mesh] OR "parasite infections" OR parsit* OR parasitic OR parasitical OR parásit* OR TORCH OR "Toxoplasmosis"[Mesh] OR toxoplasm* OR "Syphilis"[Mesh] OR sífilis OR syphilis OR "Rubella"[Mesh] OR rubéol* OR rubel* OR "Cytomegalovirus"[Mesh] OR citomegalovirus OR cytomegalovirus OR cytomégalovirus OR "Herpes Simplex"[Mesh] OR herpes OR herp* OR "Simplexvirus"[Mesh] OR "Trematode Infections"[Mesh] OR tremat* OR trématode* OR "Filariasis"[Mesh] OR Filari* OR "Onchocerciasis"[Mesh] OR Onchocerciasis OR Oncocercos* OR "Schistosoma"[Mesh] OR schisto* OR "Helminths"[Mesh] OR helmin* OR "Rabies"[Mesh] OR rabi* OR raiva OR rage OR "Trachoma"[Mesh] OR trachom* OR tracoma OR "Yaws"[Mesh] OR yaws OR bouba OR pian OR "Leprosy"[Mesh] OR leprosy OR lepra OR lèpre OR "Chagas disease"[Mesh] OR chagas OR "Leishmaniasis"[Mesh] OR leishmani* OR "Taeniasis"[Mesh] OR Taenia* OR "Echinococcus"[Mesh] OR echinoc* OR échinoc* OR equinococo OR "Neurocysticercosis"[Mesh] OR "Neglected Diseases"[Mesh] OR "neglected tropical" OR denv OR dengue OR "Dengue Virus"[Mesh] OR chikv OR chikungunya OR "Chikungunya Virus"[Mesh] OR wnv OR "west nile virus" OR "West Nile Virus"[Mesh] OR yfv OR "Yellow fever virus" OR "Yellow fever Virus"[Mesh] OR "Encephalitis Virus, Japanese"[Mesh] OR "Japanese Encephalitis Virus" OR jev OR "Encephalitis virus, St. Louis "[Mesh] OR "St. Louis encephalitis virus" OR slev OR "Kunjin virus" OR "Murray Valley encephalitis virus" OR mvev OR "Usutu virus"[Mesh] OR "Usutu virus" OR usuv OR "Encephalitis Viruses, Tick-Borne"[Mesh] OR "Tick-Borne Encephalitis Viruses" OR tbev OR "Rift Valley fever virus"[Mesh] OR "Rift Valley fever virus" OR rvfv OR OR hiv OR "human immunodeficiency virus" OR "virus da imunodeficiencia adquirida" OR "virus da imunodeficiencia humana" OR "virus da inmunodeficiencia adquirida" OR "virus da inmunodeficiencia humana" OR "HIV"[Mesh] OR AIDS OR "Respiratory Syncytial Viruses"[Mesh] OR "respiratory viral infcetion" OR RSV OR "influenza,human"[Mesh] OR influenza OR "Adenovirus Infections, Human"[Mesh] OR Adenov* OR adénovirus OR "Enterovirus"[Mesh] OR enterov* OR entérovirus OR "Tuberculosis"[Mesh] OR Tuberculos* OR astrovirus OR "Norovirus"[Mesh] OR norovirus OR "Rotavirus Infections"[Mesh] OR rotavirus OR "Giardiasis"[Mesh] OR giardias* OR giard* OR "Amebiasis" OR ameb* OR amoebiasis OR amibiase)

Web of Science:

(zikv OR zika OR "Zika Virus"[Mesh] OR "Zika Virus Infection"[Mesh]) AND (coinfec* OR (co infec*) OR co-infec* OR Coinfection[Mesh] OR "Bacterial Infections"[Mesh] OR bacterial* OR bacteria* OR bacteri* OR bactéri* OR "Parasitic Diseases"[Mesh] OR "parasite infections" OR parsit* OR parasitic OR parasitical OR parásit* OR TORCH OR "Toxoplasmosis"[Mesh] OR

Lobkowicz L, *et al. BMJ Global Health* 2020; 5:e002350. doi: 10.1136/bmjgh-2020-002350

toxoplasm* OR "Syphilis"[Mesh] OR sífilis OR syphilis OR Rubella[Mesh] OR rubéol* OR rubel* OR Cytomegalovirus[Mesh] OR citomegalovirus OR cytomegalovirus OR cytomégalovirus OR "Herpes Simplex"[Mesh] OR herpes OR herp* OR Simplexvirus[Mesh] OR "Trematode Infections"[Mesh] OR tremat* OR trématode* OR Filariasis[Mesh] OR Filari* OR Onchocerciasis[Mesh] OR Onchocerciasis OR Oncocercos* OR Schistosoma[Mesh] OR schisto* OR Helminths[Mesh] OR helmin* OR Rabies[Mesh] OR rabi* OR raiva OR rage OR Trachoma[Mesh] OR trachom* OR tracoma OR Yaws[Mesh] OR yaws OR bouba OR pian OR Leprosy[Mesh] OR leprosy OR lepra OR lèpre OR "Chagas disease"[Mesh] OR chagas OR Leishmaniasis[Mesh] OR leishmani* OR Taeniasis[Mesh] OR Taenia* OR Echinococcus[Mesh] OR echinoc* OR échinoc* OR equinococo OR Neurocysticercosis[Mesh] OR Neglected Diseases[Mesh] OR "neglected tropical" OR denv OR dengue OR "Dengue Virus"[Mesh] OR chikv OR chikungunya OR "Chikungunya Virus"[Mesh] OR wnv OR "west nile virus" OR "West Nile Virus"[Mesh] OR yfv OR "Yellow fever virus" OR "Yellow fever Virus"[Mesh] OR "Encephalitis Virus, Japanese"[Mesh] OR "Japanese Encephalitis Virus" OR jev OR "Encephalitis virus, St. Louis "[Mesh] OR "St. Louis encephalitis virus" OR slev OR "Kunjin virus" OR "Murray Valley encephalitis virus" OR mvev OR "Usutu virus"[Mesh] OR "Usutu virus" OR usuv OR "Encephalitis Viruses, Tick-Borne"[Mesh] OR "Tick-Borne Encephalitis Viruses" OR tbev OR "Rift Valley fever virus"[Mesh] OR "Rift Valley fever virus" OR rvfv OR hiv OR "human immunodeficiency virus" OR "virus da imunodeficiencia adquirida" OR "virus da imunodeficiencia humana" OR "virus da inmunodeficiencia adquirida" OR "virus da inmunodeficiencia humana" OR HIV[Mesh] OR AIDS OR "Respiratory Syncytial Viruses"[Mesh] OR "respiratory viral infection" OR RSV OR "influenza,human"[Mesh] OR influenza OR "Adenovirus Infections, Human"[Mesh] OR Adenov* OR adénovirus OR Enterovirus[Mesh] OR enterov* OR entérovirus OR Tuberculosis[Mesh] OR Tuberculos* OR astrovirus OR Norovirus[Mesh] OR norovirus OR "Rotavirus Infections"[Mesh] OR rotavirus OR Giardiasis[Mesh] OR giardias* OR giard* OR Amebiasis OR ameb* OR amoebiasis OR amibiase)

Embase:

(zikv OR zika) AND (coinfec* OR "co infection" OR "co infections" OR (co-infec*) OR bacterial* OR bacteria* OR bacteri* OR bacteri* OR "parasite infections" OR parsit* OR parasitic OR parasitical OR parasit* OR TORCH OR toxoplasm* OR sifilis OR syphilis OR rubeol* OR rubel* OR citomegalovirus OR cytomegalovirus OR cytomegalovirus OR herpes OR herp* OR tremat* OR trematode* OR Filari* OR Onchocerciasis OR Oncocercos* OR schisto*OR helmin* OR rabi* OR raiva OR rage OR trachom* OR tracoma OR yaws OR bouba OR pian OR leprosy OR lepra OR lepre OR chagas OR leishmani* OR Taenia* OR echinoc* OR echinoc* OR equinococo OR "neglected tropical" OR denv OR dengue OR chikv OR chikungunya OR wnv OR "west nile virus" OR yfv OR "Yellow fever virus" OR "Japanese Encephalitis Virus" OR jev OR "St. Louis encephalitis virus" OR slev OR "Kunjin virus" OR "Murray Valley encephalitis virus" OR mvev OR "Usutu virus" OR usuv OR "Tick-Borne Encephalitis Viruses" OR tbev OR "Rift Valley fever virus" OR rvfv OR hiv OR "human immunodeficiency virus" OR "virus da imunodeficiencia adquirida" OR "virus da imunodeficiencia humana" OR "virus da inmunodeficiencia adquirida" OR "virus da inmunodeficiencia humana" OR AIDS OR "respiratory viral infection"

OR RSV OR influenza OR Adenov* OR adenovirus OR enterov* OR enterovirus OR Tuberculos* OR astrovirus OR norovirus OR rotavirus OR giardias* OR giard* OR "Amebiasis" OR ameb* OR amoebiasis OR amibiase)

LILACs:

((tw:(zikv)) OR (tw:(zika))) AND ((tw:(coinfec*)) OR (tw:(co-infec*)) OR (tw:(bacterial*)) OR (tw:(bacteria*)) OR (tw:(bacteri*)) OR (tw:("parasite infections")) OR (tw:(parsit*)) OR (tw:(parasitic)) OR (tw:(parasitical)) OR (tw:(parásit*)) OR (tw:(TORCH)) OR (tw:(toxoplasm*)) OR (tw:(sífilis)) OR (tw:(syphilis)) OR (tw:(rubéol*)) OR (tw:(rubel*)) OR (tw:(citomegalovirus)) OR (tw:(cytomegalovirus)) OR (tw:(cytomégalovirus)) OR (tw:(herpes)) OR (tw:(herp*)) OR (tw:(tremat*)) OR (tw:(trématode*)) OR (tw:(Filari*)) OR (tw:(Onchocerciasis)) OR (tw:(Oncocercos*)) OR (tw:(schisto*)) OR (tw:(helmin*)) OR (tw:(rabi*)) OR (tw:(raiva)) OR (tw:(rage)) OR (tw:(trachom*)) OR (tw:(tracoma)) OR (tw:(yaws)) OR (tw:(bouba)) OR (tw:(pian)) OR (tw:(leprosy)) OR (tw:(lepra)) OR (tw:(lèpre)) OR (tw:(chagas)) OR (tw:(leishmani*)) OR (tw:(Taenia*)) OR (tw:(echinoc*)) OR (tw:(échinoc)) OR (tw:(equinococo)) OR (tw:("neglected tropical")) OR (tw:(denv)) OR (tw:(dengue)) OR (tw:(chikv)) OR (tw:(chikungunya)) OR (tw:(wnv)) OR (tw:("west nile virus")) OR (tw:(yfv)) OR (tw:("Yellow fever virus")) OR (tw:("Japanese Encephalitis Virus")) OR (tw:(jev)) OR (tw:("St. Louis encephalitis virus")) OR (tw:(slev)) OR (tw:("Kunjin virus")) OR (tw:("Murray Valley encephalitis virus")) OR (tw:(mvev)) OR (tw:("Usutu virus")) OR (tw:(usuv)) OR (tw:("Tick-Borne Encephalitis Viruses")) OR (tw:(tbev)) OR (tw:("Rift Valley fever virus")) OR (tw:(rvfv)) OR (tw:(hiv)) OR (tw:(aids)) OR (tw:("human immunodeficiency virus")) OR (tw:("virus da imunodeficiencia adquirida")) OR (tw:("virus da imunodeficiencia humana")) OR (tw:("virus da inmunodeficiencia adquirida")) OR (tw:("virus da inmunodeficiencia humana")) OR (tw:("respiratory viral infection")) OR (tw:(rsv)) OR (tw:(influenza)) OR (tw:(Adenov*)) OR (tw:(adénovirus)) OR (tw:(enterov*)) OR (tw:(entérovirus)) OR (tw:(Tuberculos*)) OR (tw:(astrovirus)) OR (tw:(norovirus)) OR (tw:(rotavirus)) OR (tw:(giardias*)) OR (tw:(giard*)) OR (tw:("Amebiasis")) OR (tw:(ameb*)) OR (tw:(amoebiasis)) OR (tw:(amibiase)))

Lobkowicz L, *et al. BMJ Global Health* 2020; 5:e002350. doi: 10.1136/bmjgh-2020-002350

Supplementary Appendix 2. ***Oxford Centre for Evidence-based Medicine – Level of Evidence. Table replicated from: https://www.cebm.net/2009/06/oxford-centre-evidence-based-medicine-levels-evidence-march-2009/*** **[11].**

Level	Therapy / Prevention, Aetiology / Harm	Prognosis	Diagnosis	Differential diagnosis / symptom prevalence study	Economic and decision analyses
**1a**	SR (with homogeneity*) of RCTs	SR (with homogeneity*) of inception cohort studies; CDR" validated in different populations	SR (with homogeneity*) of Level 1 diagnostic studies; CDR" with 1b studies from different clinical centres	SR (with homogeneity*) of prospective cohort studies	SR (with homogeneity*) of Level 1 economic studies
**1b**	Individual RCT (with narrow Confidence Interval"¡)	Individual inception cohort study with > 80% follow-up; CDR" validated in a single population	Validating** cohort study with good" " " reference standards; or CDR" tested within one clinical centre	Prospective cohort study with good follow-up****	Analysis based on clinically sensible costs or alternatives; systematic review(s) of the evidence; and including multi-way sensitivity analyses
**1c**	All or none§	All or none case series	Absolute SpPins and SnNouts" "	All or none case series	Absolute better-value or worse-value analyses " " " "
**2a**	SR (with homogeneity*) of cohort studies	SR (with homogeneity*) of either retrospective cohort studies or untreated control groups in RCTs	SR (with homogeneity*) of Level >2 diagnostic studies	SR (with homogeneity*) of 2b and better studies	SR (with homogeneity*) of Level >2 economic studies
**2b**	Individual cohort study (including low quality RCT; e.g., <80% follow-up)	Retrospective cohort study or follow-up of untreated control patients in an RCT; Derivation of CDR" or validated on split-sample§§§ only	Exploratory** cohort study with good" " " reference standards; CDR" after derivation, or validated only on split-sample§§§ or databases	Retrospective cohort study, or poor follow-up	Analysis based on clinically sensible costs or alternatives; limited review(s) of the evidence, or single studies; and including multi-way sensitivity analyses
**2c**	"Outcomes" Research; Ecological studies	"Outcomes" Research		Ecological studies	Audit or outcomes research

**3a**	SR (with homogeneity*) of case-control studies		SR (with homogeneity*) of 3b and better studies	SR (with homogeneity*) of 3b and better studies	SR (with homogeneity*) of 3b and better studies
**3b**	Individual Case-Control Study		Non-consecutive study; or without consistently applied reference standards	Non-consecutive cohort study, or very limited population	Analysis based on limited alternatives or costs, poor quality estimates of data, but including sensitivity analyses incorporating clinically sensible variations.
**4**	Case series (and poor quality cohort and case-control studies§§)	Case series (and poor quality prognostic cohort studies***)	Case-control study, poor or non-independent reference standard	Case series or superseded reference standards	Analysis with no sensitivity analysis
**5**	Expert opinion without explicit critical appraisal, or based on physiology, bench research or "first principles"	Expert opinion without explicit critical appraisal, or based on physiology, bench research or "first principles"	Expert opinion without explicit critical appraisal, or based on physiology, bench research or "first principles"	Expert opinion without explicit critical appraisal, or based on physiology, bench research or "first principles"	Expert opinion without explicit critical appraisal, or based on economic theory or "first principles"

* By homogeneity we mean a systematic review that is free of worrisome variations (heterogeneity) in the directions and degrees of results between individual studies. Not all systematic reviews with statistically significant heterogeneity need be worrisome, and not all worrisome heterogeneity need be statistically significant. As noted above, studies displaying worrisome heterogeneity should be tagged with a "-" at the end of their designated level.

" Clinical Decision Rule. (These are algorithms or scoring systems that lead to a prognostic estimation or a diagnostic category.)

"¡ See note above for advice on how to understand, rate and use trials or other studies with wide confidence intervals.

§ Met when all patients died before the Rx became available, but some now survive on it; or when some patients died before the Rx became available, but none now die on it.

§§ By poor quality cohort study we mean one that failed to clearly define comparison groups and/or failed to measure exposures and outcomes in the same (preferably blinded), objective way in both exposed and non-exposed individuals and/or failed to identify or appropriately control known confounders and/or failed to carry out a sufficiently long and complete follow-up of patients. By poor quality case-control study we mean one that failed to clearly define comparison

groups and/or failed to measure exposures and outcomes in the same (preferably blinded), objective way in both cases and controls and/or failed to identify or appropriately control known confounders.

§§§ Split-sample validation is achieved by collecting all the information in a single tranche, then artificially dividing this into "derivation" and "validation" samples.

" " An "Absolute SpPin" is a diagnostic finding whose Specificity is so high that a Positive result rules-in the diagnosis. An "Absolute SnNout" is a diagnostic finding whose Sensitivity is so high that a Negative result rules-out the diagnosis.

"¡"¡ Good, better, bad and worse refer to the comparisons between treatments in terms of their clinical risks and benefits.

" " " Good reference standards are independent of the test, and applied blindly or objectively to applied to all patients. Poor reference standards are haphazardly applied, but still independent of the test. Use of a non-independent reference standard (where the 'test' is included in the 'reference', or where the 'testing' affects the 'reference') implies a level 4 study.

" " " " Better-value treatments are clearly as good but cheaper, or better at the same or reduced cost. Worse-value treatments are as good and more expensive, or worse and the equally or more expensive.

** Validating studies test the quality of a specific diagnostic test, based on prior evidence. An exploratory study collects information and trawls the data (e.g. using a regression analysis) to find which factors are 'significant'.

*** By poor quality prognostic cohort study we mean one in which sampling was biased in favour of patients who already had the target outcome, or the measurement of outcomes was accomplished in <80% of study patients, or outcomes were determined in an unblinded, non-objective way, or there was no correction for confounding factors.

**** Good follow-up in a differential diagnosis study is >80%, with adequate time for alternative diagnoses to emerge (for example 1-6 months acute, 1 - 5 years chronic)

Lobkowicz L, *et al. BMJ Global Health* 2020; 5:e002350. doi: 10.1136/bmjgh-2020-002350
